# Culture media selection and feeding strategy for high titer production of a lentiviral vector by stable producer clones cultivated at high cell density

**DOI:** 10.1007/s00449-022-02737-5

**Published:** 2022-06-27

**Authors:** Chun Fang Shen, Sonia Tremblay, Catherine Sabourin-Poirier, Elodie Burney, Sophie Broussau, Aziza Manceur, Anja Rodenbrock, Robert Voyer, Martin Loignon, Sven Ansorge, Rénald Gilbert

**Affiliations:** Human Health Therapeutics Research Centre, National Research Council of Canada, Montreal, Canada

**Keywords:** Lentiviral vector, Stable producer clone, Culture medium optimization, Fed-batch, High cell density

## Abstract

The growing interest in the use of lentiviral vectors (LVs) for various applications has created a strong demand for large quantities of vectors. To meet the increased demand, we developed a high cell density culture process for production of LV using stable producer clones generated from HEK293 cells, and improved volumetric LV productivity by up to fivefold, reaching a high titer of 8.2 × 10^7^ TU/mL. However, culture media selection and feeding strategy development were not straightforward. The stable producer clone either did not grow or grow to lower cell density in majority of six commercial HEK293 media selected from four manufacturers, although its parental cell line, HEK293 cell, grows robustly in these media. In addition, the LV productivity was only improved up to 53% by increasing cell density from 1 × 10^6^ and 3.8 × 10^6^ cells/mL at induction in batch cultures using two identified top performance media, even these two media supported the clone growth to 5.7 × 10^6^ and 8.1 × 10^6^ cells/mL, respectively. A combination of media and feed from different companies was required to provide diverse nutrients and generate synergetic effect, which supported the clone growing to a higher cell density of 11 × 10^6^ cells/mL and also increasing LV productivity by up to fivefold. This study illustrates that culture media selection and feeding strategy development for a new clone or cell line can be a complex process, due to variable nutritional requirements of a new clone. A combination of diversified culture media and feed provides a broader nutrients and could be used as one fast approach to dramatically improve process performance.

## Introduction

Lentiviral vectors (LVs) have emerged to be valuable tools as gene transfer vehicles because of their ability to integrate their genomes into the chromosomes of dividing and non-dividing cells for stable transgene expression. Their potential for the integration and long-term expression of therapeutic genes renders them an interesting tool for gene and cell therapy interventions [1], such as to introduce genes into mature T cells to generate immunity to cancer through the delivery of chimeric antigen receptors (CARs) or cloned T cell receptors [2]. LV vectors have been used successfully in clinical trials for the treatment of several diseases such as primary immunodeficiencies [3] and Parkinson’s disease [4]. The first lentivirally transduced cellular therapy, tisagenlecleucel (CTL019, Kymriah), was approved in the United States in August of 2017 for the treatment of pediatric and young adult patients with acute lymphoblastic leukemia [5]. As interest in the use of lentiviral vectors for various applications continues to grow, further development of cost-effective and robust process for mass production of LVs is needed [6–8].

LVs are produced in the following two different ways: by transient transfection of human cells or by development of stable vector packaging cell lines [9, 10]. LV production by multi-plasmid transient transfection has been the most widely used technique for the generation of LV stocks in academic laboratories and industry for both research and clinical gene therapy applications since it constitutes a faster, simpler, and more versatile approach [11–13]. There are extensive reports of scaling up production using transient transfection by examining media formulations, transfection agents, plasmid design, production modes, downstream processing, and bioreactor configurations. Titers from 10^6^ to 2 × 10^8^ TU/mL have been reported, and the production has been scaled up to 200L suspension culture bioreactor [6, 7, 10, 14]. One drawback of the transfection procedure is the amount of plasmid DNA required and expensive transfection reagents used to reach high transfection efficiency, which consequently makes the production process extremely costly at industrial scales. In addition, it is still difficult to transfect cells at high cell density, limiting the vector productivity of the transfection process.

Compared with transient production, production of LVs using packaging cell lines is advantageous, because it requires only for a single DNA that carries the gene of interest flanked by the two human immunodeficiency virus long terminal repeats (the transfer vector). Stable LV manufacturing is a desirable approach for gene therapy to reduce the production costs and increase the overall safety and reproducibility [15]. Although many stable packaging cell lines have been developed as summarized in review articles [7, 9], a common feature of these packaging cell lines is the reliance on adherent, serum-dependent cells, which may not be amenable to commercial-scale cGMP production. Most of the developmental work was conducted in multi-well plates or T-flask settings with adherent cultures and with LV titers ranging from 10^6^ to 5 × 10^7^ TU/mL. There has been less extensive progress to date in the process development for LVs production using stable cell lines, especially with suspension adapted packaging cell lines [10, 16].

Our research group started exploring the feasibility of using packaging cell lines as a technology for production of LVs over a decade ago, when a packaging cell line (293SF-PacLV) was developed and used to produce lentiviral vectors (LVs) in serum-free suspension bioreactor batch cultures [17]. Recently, more stable producer cell lines have been developed and studied for the possibility of high titer LV production. A titer of 6 × 10^6^ TU/mL was achieved in batch culture. The LV titer was improved significantly, reaching a cumulative total yield of 8 × 10^7^ TU/mL in bioreactor perfusion process [18]. In the perfusion process, a feeding strategy of continuously replacing spent medium with fresh medium was employed to supply additional amount of nutrients to support cell growth and virus production at higher cell densities in order to achieve higher titer production. As a result, substantial improvement of LV productivity was achieved in the perfusion culture when comparing to the titer obtained in batch culture. However, the perfusion process also shows some drawbacks. For example, the operation of perfusion process is costly and more complicated than the batch or fed-batch process. In addition, the setup and validation of perfusion process is complex.

Batch and fed-batch cultures are simple and most commonly used processes for production of biologics in biotechnology industries. Fed-batch cell culture protocols are the most widely used culture operating mode for high volumetric productivity of many biopharmaceuticals, especially monoclonal antibodies. In the fed-batch process, a basal medium supports initial cell growth, while a feed medium prevents depletion of nutrients, sustains the growth phase to high density, and enhances the production [19]. Processes at high cell density allow the use of compact bioreactors with high volumetric production rates, and therefore also reduce the manufacturing cost. Although fed-batch processes have high potential, development of fed-batch for virus production has only sporadically been reported [20, 21], which might be partially due to the complexity of nutritional requirements in the biphasic viral and vector production process [22]. The objective of this study was to develop simpler processes such as batch or fed-batch process for high titer production of LVs at higher cell density through using better nutrient balanced culture media and/or developing feeding strategy. The balanced media and feeding strategy would improve nutrient supplies to meet different nutritional requirements during the phases of cell growth and virus production. The outcome of this study provided a simple and low-cost high cell density process for high titer production of LVs and might eventually help the design of culture media in combination of feed which not only sustain the growth of cells to high density but also support the production of LV by this increased biomass.

## Materials and methods

### Cell line, media and cell culture

The stable producer clones (clones P/cSIN/92 and P/SIN/18) used in the current study were generated using packaging cells #29-6 (a derivative of HEK293 cell) and described before [17]. Briefly, the clone P/cSIN/92 was generated by transducing the packaging cells with a conditional self-inactivating LV produced by transfecting the packaging cells with pTet07-CSII-CMVGFPq. The resulting pool was then cloned by limiting dilution. To generate clone P/SIN/18, the packaging cells were co-transfected with plasmid pCSIICMV5-GFP which encodes a self-inactivating LV, and a plasmid pCDNA6-hisA (Life Technologies) encoding the resistance for blasticidin (InvivoGen). A pool of blasticidin-resistant cells was generated and then cloned by limiting dilution.

Both clones P/cSIN/92 and P/SIN/18 were adapted and grown in culture media at 20 mL culture volume in 125 mL plastic shake flasks (Corning, NY) at 37 °C and 5% CO_2_ with agitation of 120 rpm in a humidified incubator and maintained in that medium for at least 2 weeks before initiating a batch culture experiment. Cells were subcultured to densities of between 0.2 and 0.25 × 10^6^ cell/mL three times a week. The culture was sampled regularly for cell counts and other analyses as needed. The cells were discarded after 2 months in culture.

The media and feeds used in this study included the following: HyCell™ TransFx-H, SFM4transfx 293 and Cell Boost 5 (all from Cytiva, Logan, UT), BalanCD HEK293 (FUJIFILM Irvine Scientific, Santa Ana, CA), FreeStyle F17 (Gibco, Thermo Fisher Scientific, Waltham, MA), HEK TF, HEK GM and HEK FS (all from Xell AG, Bielefeld, Germany). These media and feeds were mainly designed for HEK293 cell culture and might be also choices for clones P/cSIN/92 and P/SIN/18 as both of them were generated from HEK293 cell. Top performance media selected after media testing were employed as basal media for development of high cell density culture process through feeding commercial feed to the culture. Processes developed with two commercial feeds, Cell Boost 5 (CB5) and HEK FS feeds, were described as follows: Culture was seeded at 0.20–0.25 × 10^6^ cells/mL in HyCell TransFx-H, and fed with 3% and 5% (v/v) CB5 when the cultures reached respective cell densities of about 3 × 10^6^ cells/mL and 5 × 10^6^ cells/mL. The CB5 feed was prepared at a concentration of 35 g/L according to the manufacturer’s instruction. In other processes using HEK FS feed, the cells were seeded at 0.20–0.25 × 10^6^ cells/mL in several different media, including HyCell TransFx-H, HEK TF and a mixture of 50% HyCell TransFx-H + 50% HEK TF. Each culture was then fed with HEK FS at 4% (v/v), 6% and 6% at day 3, 4, and 6 respectively, during the time course of the culture process.

### Production of LV by induction of stable producer clones P/cSIN/92 and P/SIN/18

#### Production of LV in batch culture without nutrient supplements during the cell growth or virus production

**Fig. 1:**
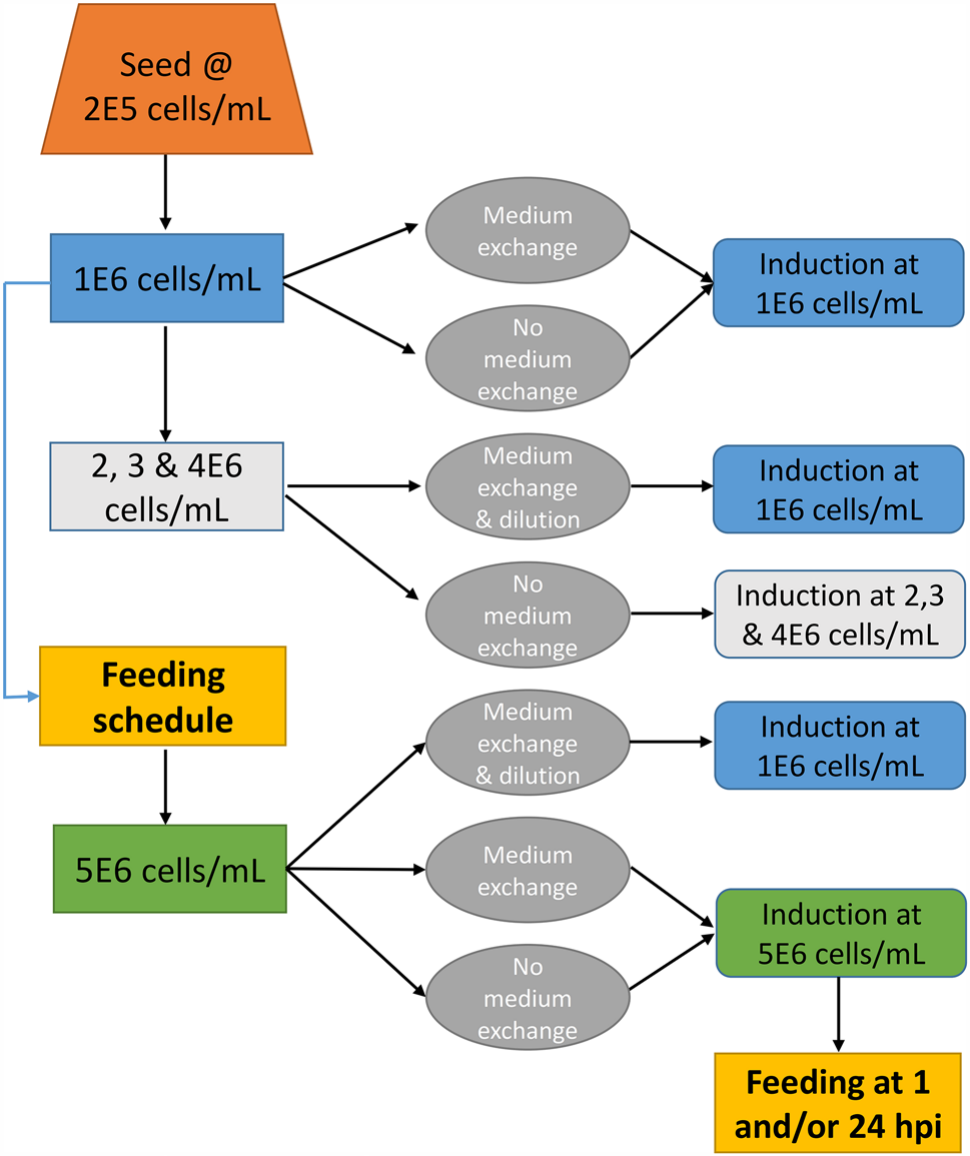
Scheme of culture conditions designed to study the production of lentiviral vector using clone P/cSIN/92. The clone was first cultivated in HyCell TransFx-H to examine volumetric virus productivity in batch cultures induced at cell density of 1, 2, 3 and 4E6 cells/mL and without medium exchange. The cultures grown to different concentrations were also taken out, centrifuged and then resuspended to fresh media at 1E6 cells/mL (medium exchange & dilution) before induction to assess specific virus productivity of cell. The clone was also, respectively, grown in three culture media, HyCell TransFx-H, HEK TF and a mixture of 50% HEK TF and 50% HyCell TransFx-H, and fed with Cellboost 5 or HEK FS feed to a cell density of 5E6 cells/mL and induced with or without medium exchange prior to induction. The induced culture was then fed at 1 and/or 24 hpi as needed by experimental design to investigate the virus production in fed-batch culture induced at high cell density (5E6 cells/mL)

Cells were seeded at 0.20–0.25 × 10^6^ cells/mL and amplified in 100–150 mL of each medium in 500 mL shake flasks. When cells reached the desired density between 1 × 10^6^ cells/mL to 4 × 10^6^ cells/mL, cells were subjected or not to a medium exchange step prior to induction as illustrated in Fig. 1. For the medium exchange, the appropriate volume of cells was centrifuged at 300 g for 5 min at room temperature, then resuspended in the initial volume of fresh medium. The resuspended cells were then aliquoted to 2 × 20 mL in duplicate 125 mL shake flasks and induced for LV production. Induction of the culture was done through the addition of 1 µg/mL of doxycycline and 30 µg/mL of cumate (final concentration). This concentration is appropriate for induction of cultures with a cell density up to 5 × 10^6^ cells/mL based on a previous dose-dependent study. The induced cultures were sampled regularly and centrifuged at 300 g for 5 min. The supernatant was collected, aliquoted and stored at -80 °C for subsequent analysis.

#### Production of LV in high density culture with nutrient supplements (feeding) during the cell growth and/or virus production

In the cell culture process developed using HyCell TransFx-H medium and CB5 feed, culture was seeded at 0.20–0.25 × 10^6^ cells/mL and then grown to different cell densities according to the feeding strategy described previously. When cells reached the desired density such as 1, 2, 3 and 5 × 10^6^ cells/mL, a portion of culture was withdrawn from the shake flasks, and subjected or not to a medium exchange step prior to induction. Some cultures were fed with 3 to 5% CB5 feed or supplemented with other nutrients (such as insulin, yeast extract) before the induction. The induced cultures were sampled at 72 hpi for LV quantification.

Cell growth and culture preparation for LV production in other cultures using HEK FS as feed were also illustrated in Fig. 1. Cells were seeded at 0.20–0.25 × 10^6^ cells/mL in HyCell TransFx-H, HEK TF and a mixture of 50% HyCell TransFx-H + 50% HEK TF, respectively, and fed with HEK FS accordingly to the feeding schedule developed. When cells reached the desired density of 1 and 5 × 10^6^ cells/mL, a portion of culture was withdrawn from the shake flasks and prepared according to Fig. 1 for induction in duplicate. Induced cultures were sampled at 72 hpi for LV quantification.

### Analytical methods

Total and viable cells were measured by incubating culture samples with Accumax (STEMCELL Technologies Inc. Vancouver, Canada), a cell detachment solution, for 30 min to create single-cell suspensions and then followed by automatic cell count using the Cedex HiRes Analyzer (Roche Diagnostics Corporation, Indianapolis, IN). Amino acids in fresh and spent media were analyzed by a HPLC method as described before [22]. Concentration of glucose, lactate and ammonia was measured by Cedex BioAnalyzer (Roche CustomBiotech). Functional viral titer (Gene Transfer Assay, GTA titer) of samples taken from shake flask cultures was determined using a flow cytometry–based GTA as described previously [18].

## Results

### Growth of clone P/cSIN/92 in different media with or without nutrient supplement:

**Fig. 2:**
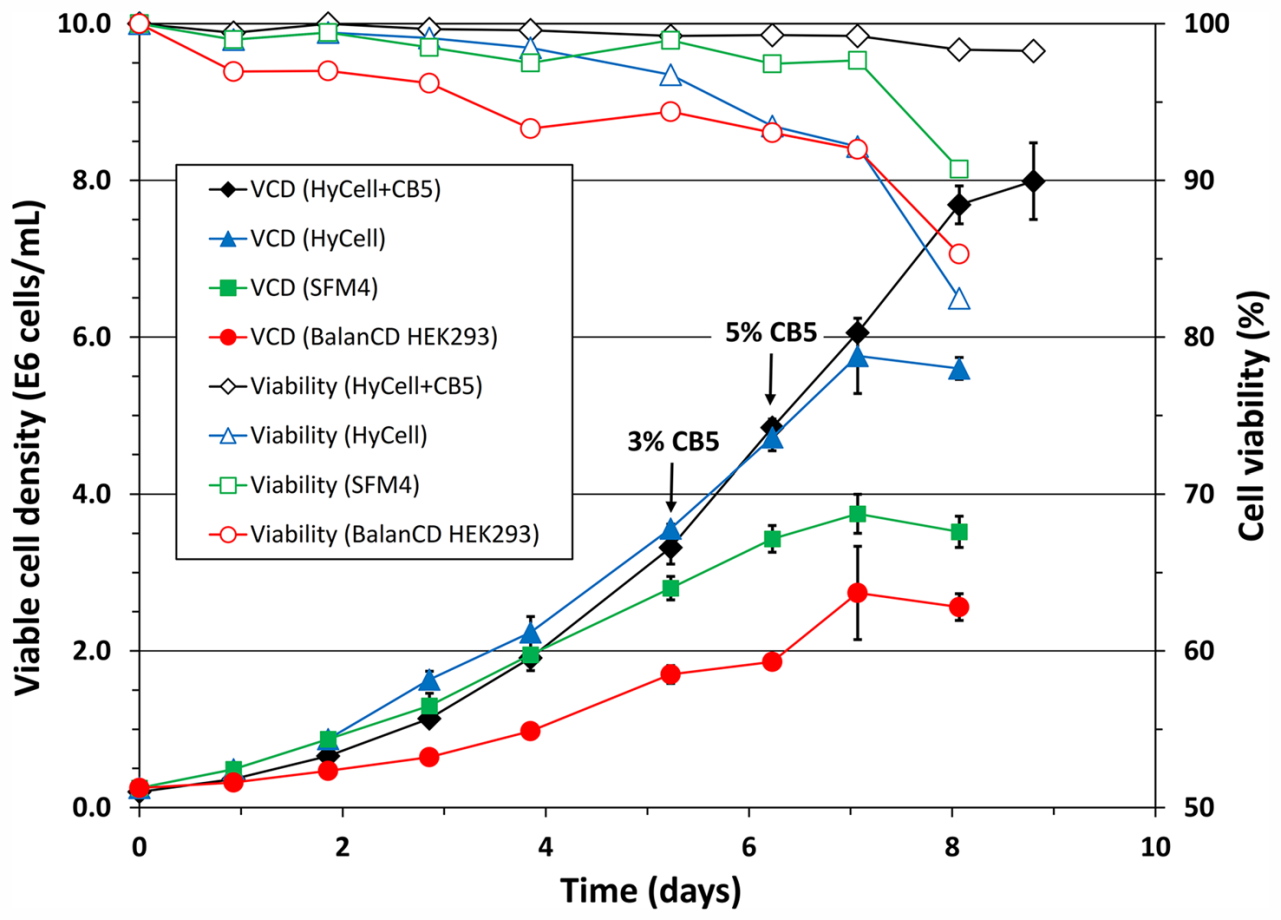
Growth curves of clone P/cSIN/92 in batch cultures, respectively, using three different culture media, namely BalanCD HEK293 (red circle), SFM4transfx 293 (SFM4, green square) and HyCell TransFx-H (HyCell, blue triangle). Feeding the HyCell TransFx-H culture (black diamond) with Cell Boost 5 (CB5) improved the maximum viable cell density. Bars represent the mean of duplicate cultures ± standard deviation (colour figure online)

**Fig. 3:**
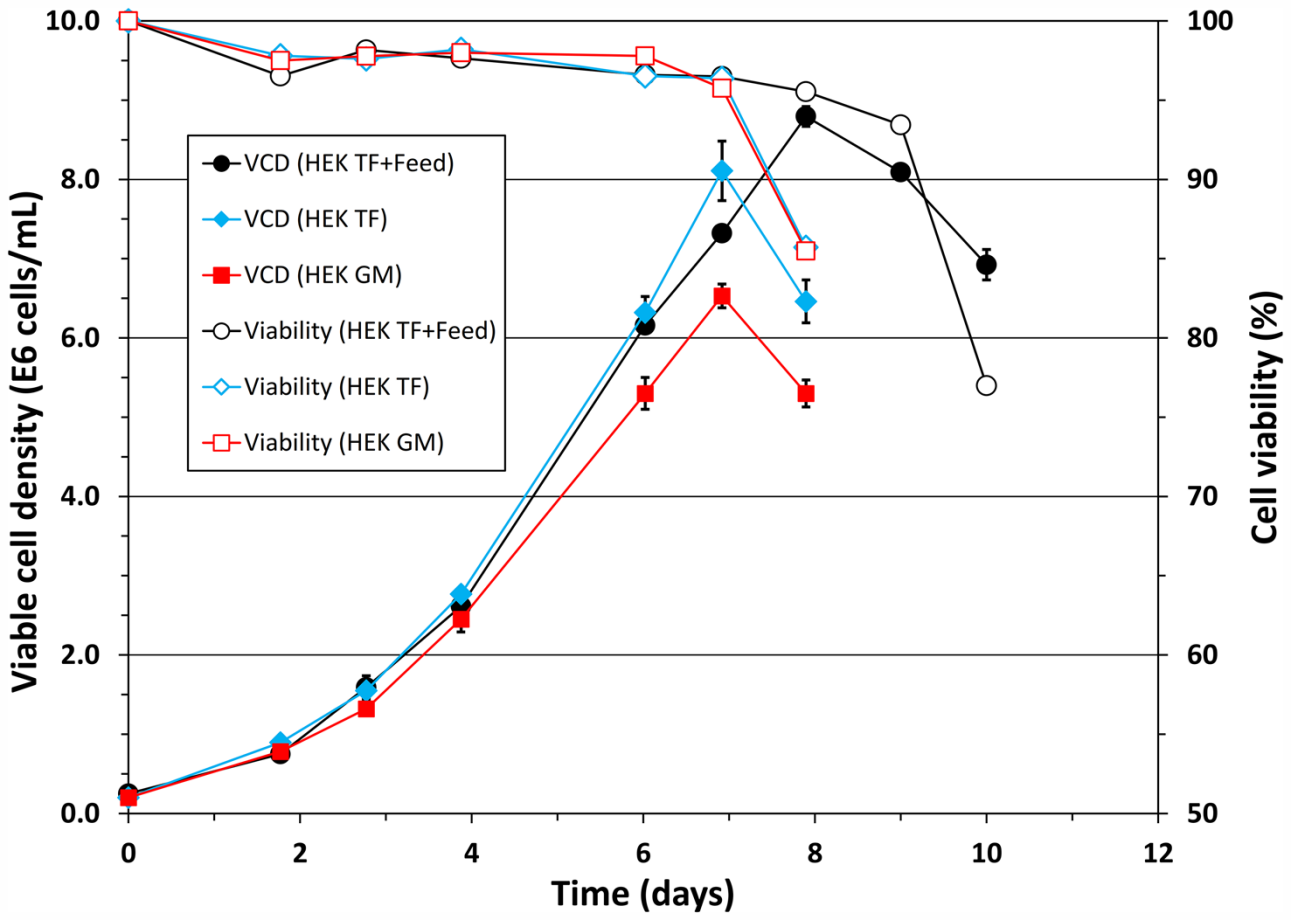
Growth curves of clone P/cSIN/92 in batch cultures using HEK TF (blue diamond) and HEK GM (red square) as growth media. Feeding the HEK TF culture (black circle) with HEK FS feed only increased the maximum cell density marginally. Bars represent the mean of duplicate cultures ± standard deviation (colour figure online)

The growth of clone P/cSIN/92 was evaluated in six commercial media with or without supplement of the two commercial feeds, CB5 and HEK FS. SFM4transfx 293 was used as a basal medium in previous studies and included in this experiment as a reference. The cell growth in FreeStyle F17 slowed down after one passage and stopped after three passages. Experimental results from other five media and two feeds are shown in Figs. 2 and 3, respectively. The data in Fig. 2 revealed that the clone only grew to a maximum viable cell density of 2.7 × 10^6^ cells/mL and 3.7 × 10^6^ cells/mL in batch culture, respectively, using BalanCD HEK293 or SFM4transfx 293. A significant higher cell density, 5.7 × 10^6^ cells/mL, was achieved when clone P/cSIN/92 was cultivated in HyCell™ TransFx-H, and a cell doubling time was 27 h during the exponential growth phase. Feeding the HyCell™ TransFx-H culture with 3% and 5% CB5 feed, respectively, on days 5 and 6 increased the maximum viable cell density to 8 × 10^6^ cells/mL.

The cell growth was promising in HEK GM and HEK TF (both media from Xell AG), respectively, reaching 6.5 and 8.1 × 10^6^ viable cells/mL in batch cultures (Fig. 3). Feeding the HEK TF culture with HEK FS feed increased the maximum viable cell density from 8.1 × 10^6^ cells/mL to 8.8 × 10^6^ cells/mL. The higher cell density (> 8 × 10^6^ cells/mL) achieved in the cultures using HyCell™ TransFx-H and CB5 or HEK TF and HEK FS feed provides opportunities to explore the induction of clone P/cSIN/92 culture at high cell density (for example 5 × 10^6^ cells/mL) for possible higher titer production of LVs. Consequentially, HyCell™ TransFx-H and CB5, HEK TF and HEK FS feed were selected for further experiments aimed to improve LV productivity.

### Production of LV in clone P/cSIN/92 cultures grown with HyCell TransFx-H or HEK TF without feeding before or after induction

**Fig. 4:**
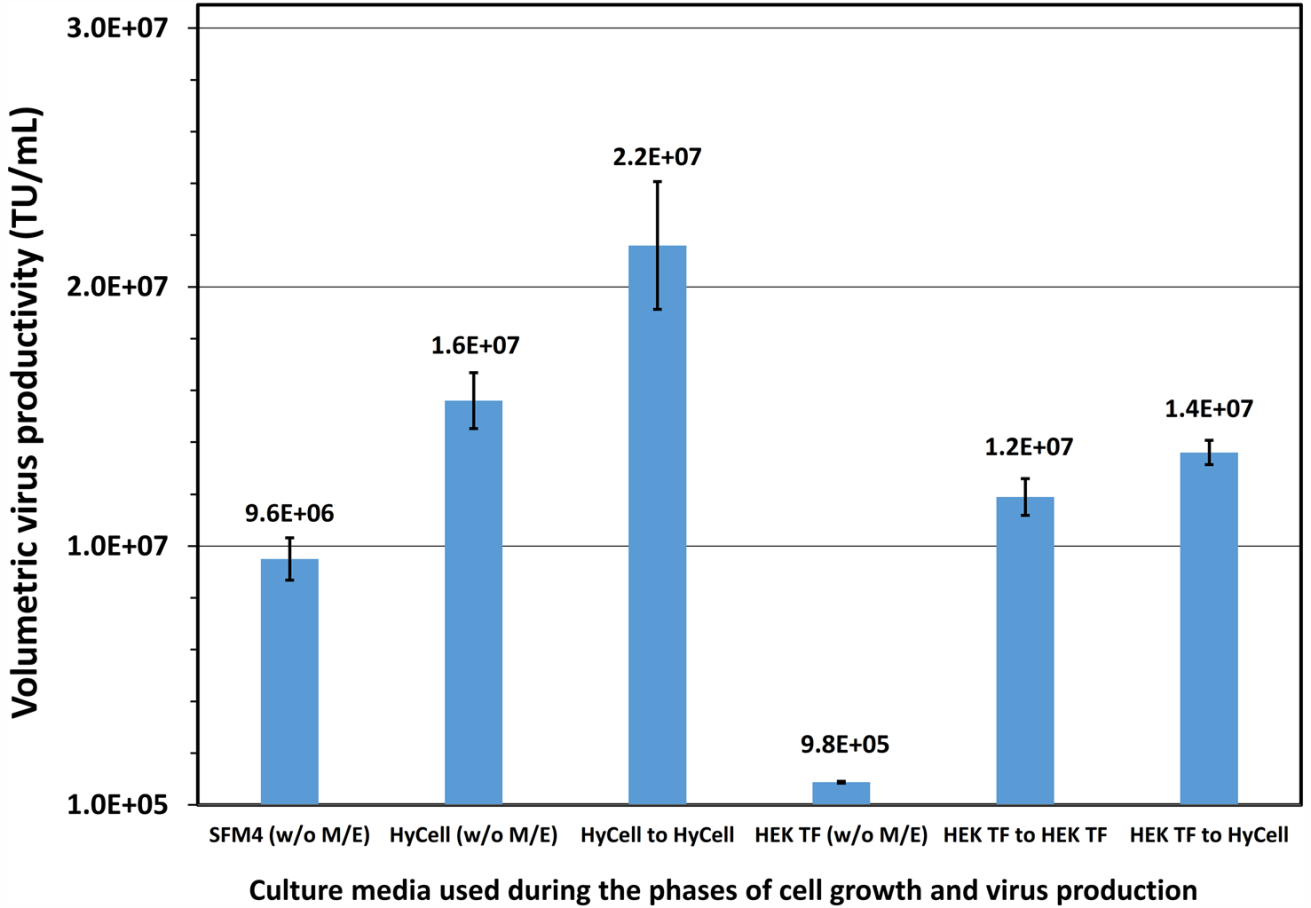
Lentiviral vector production in clone P/cSIN/92 batch culture grown in SFM4transfx 293 (SFM4), HyCell TransFx-H (HyCell) or HEK TF to a density of 1E6 cells/mL and then without medium exchange (w/o M/E) or with medium exchange to the same or different medium before induction (duplicate cultures)

Batch culture was first conducted to evaluate LV productivity in cultures grown in the two identified top performance media, HyCell TransFx-H or HEK TF, and induced at a cell density of 1 × 10^6^ cells/mL. In parallel, a culture grown in SFM4transfx 293 to 1 × 10^6^ cells/mL and induced without medium exchange was conducted as a reference. Figure 4 showed the LV productivity was very poor, at 9.75 × 10^5^ TU/mL, in the culture grown with HEK TF and induced without medium exchange. This titer was lower than the 9.6 × 10^6^ TU/mL achieved in the reference with SFM4transfx 293. A medium exchange by resuspending the cells grown in HEK TF to fresh HEK TF or HyCell TransFx-H medium before induction restored the LV production to1.2 × 10^7^ and 1.4 × 10^7^ TU/mL, respectively. In contrast, the LV production was much better in the culture grown with HyCell TransFx-H, and was at 1.57 × 10^7^ TU/mL in the culture induced without medium exchange. The titer was further improved and reached 2.17 × 10^7^ TU/mL in the culture when a medium exchange with fresh HyCell TransFx-H was carried out. This result may suggest that nutrients required in the phase of LV production were sub-optimal in the HEK TF medium, and quality of the cells grown in HEK TF was also sub-optimal for promoting the virus production, even though HEK TF supported the clone growing to high cell density. In comparison, HyCell TransFx-H is better formulated to support the LV productivity of clone P/cSIN/92 and was, therefore, used in further experiments aiming to improve LV production.

**Fig. 5:**
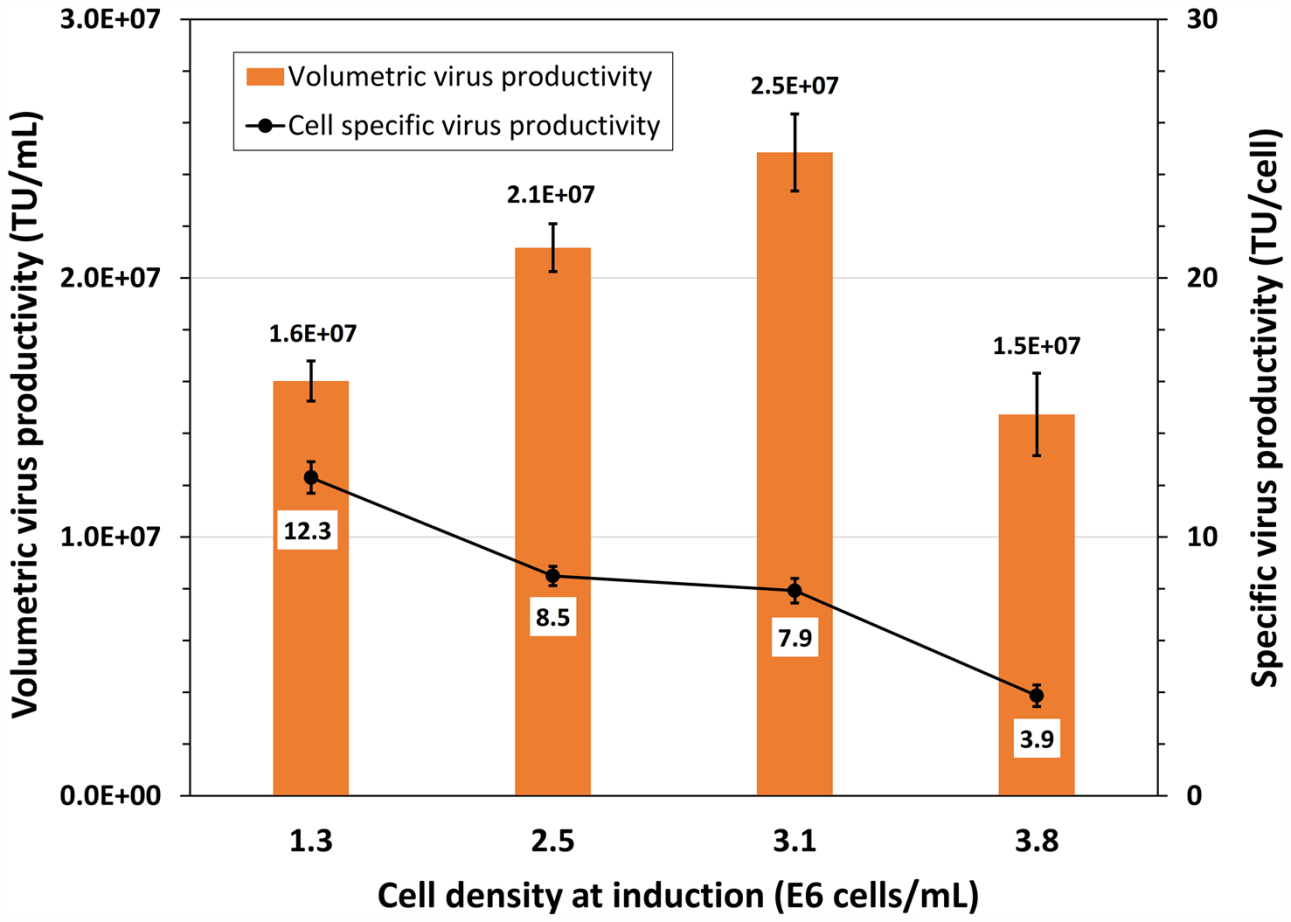
Volumetric and cell specific lentiviral vector productivity in clone P/cSIN/92 batch culture with HyCell TransFx-H medium grown to and induced at different cell densities without medium replacement. Bars represent the mean of duplicate cultures ± standard deviation

Batch culture was then explored to examine a possibility of improving LV productivity through induction of batch culture at different densities. Data in Fig. 5 reveal that the volumetric LV titer was improved from 1.6 × 10^7^ to 2.5 × 10^7^ TU/mL (or up to 53% increase) when the cell density at induction was increased from 1.3 to 3.1 × 10^6^ cells/mL. With further increase of cell density at induction to 3.8 × 10^6^ cells/mL, the virus productivity dropped to 1.5 × 10^7^ TU/mL or 92% of that obtained in the culture induced at 1.3 × 10^6^ cells/mL (Fig. 5). When converting the above volumetric productivity into the cell specific vector productivity, which is calculated by dividing the volumetric productivity by the cell density at induction, it shows that the cell specific vector productivity declined from 12.3 to 3.9 TU/mL when the cell density at induced increased from 1.3 × 10^6^ to 3.8 × 10^6^ cells/mL.

One set of experiment was conducted to examine whether the declined cell-specific vector productivity was due to change of the “quality” of clone P/cSIN/92 grown to high cell density. In this experiment, cultures grown to different cell densities were centrifuged, respectively. Cell pellet was then resuspended to fresh HyCell TransFx-H medium at a final density of 1 × 10^6^ cells/mL before induction to ensure that all cells from different density cultures were in the same culture environment (such as available nutrients) during the vector production phase. Data in Table 1 reveal that the cell-specific vector productivity was about 17 TU/cell for the cells taken from the three cultures grown to different densities, suggesting the cells prior to induction were in the same state (or “quality”) in terms of supporting the virus production. The 25.7 TU/cell for the cells from the culture at a density of 2.74 × 10^6^ cells/mL was significantly higher, which could be due to deviation of experimental conditions.

**Table 1:** Cell-specific vector productivity of clone P/cSIN/92 taken from batch cultures grown to different cell concentrations

Culture concentration (× 10^6^ cells/mL)	Cell concentration at induction (× 10^6^ cells/mL)	Average titer (TU/cell, n = 2)	SD (TU/cell)
1.63	1.0	16.5	1.9
2.74	1.0	25.7	3.5
4.06	1.0	16.8	0.3
4.60	1.0	16.9	1.1

Cultures grown to different concentrations were taken out, centrifuged and then resuspended to fresh HyCell TransFx-H medium at 1 × 10^6^ cells/mL before induction (duplicate cultures)

#### Production of LV in clone P/cSIN/92 culture grown HyCell TransFx-H in high cell density culture with nutrient supplement and/or medium exchange before induction

Several sets of experiments were dedicated to investigate if the LV productivity could be significantly increased by inducing the culture at high cell density (such as 5 × 10^6^ cells/mL) and through feeding the culture prior to and/or post the induction. The tested nutrients (feeds) included CB5 from 1 to 5%, yeast extract (Biospringer) at 2–4 g/L, insulin (10–100 µg/mL, ThermoFisher Scientific) or a combination of different nutrients. Results from these sets of experiments revealed that, when compared to the culture induced at 1 × 10^6^ cells/mL, improvement in the LV volumetric productivity was marginal except 2.2-fold increase achieved in the culture induced at 5 × 10^6^ cells/mL with a complete medium exchange before the induction (Table 2). The LV titer actually declined in the culture only fed with 3% or 5% CB. Data from additional test on the effect of CB5 supplement (not shown) suggest that CB5 feed might interfere with the induction and/or vector production.

**Table 2:** A summary of typical results of volumetric LV productivity of clone P/cSIN/92 grown in HyCell™ TransFx-H culture, fed with CB5 and/or other nutrients and with or without medium exchange before induction

Cell density at induction (cells/mL)	Medium exchange	Feed	Titer (TU/mL)	SD (TU/mL)
1 × 10^6^	No	–	1.53E + 07	1.2E + 06
3 × 10^6^	No	3% cell boost 5	1.28E + 07	1.5E + 06
5 × 10^6^	No	–	1.09E + 07	5.0E + 05
5 × 10^6^	No	50 µg/mL insulin	1.40E + 07	1.5E + 06
5 × 10^6^	No	2% cell boost 5 + 2% yeast extract	1.85E + 07	2.0E + 06
5 × 10^6^	No	5% cell boost 5	8.0E + 06	8.0E + 05
5 × 10^6^	Yes	–	3.38E + 07	2.0E + 06

*SD* standard deviation from duplicate infected cultures

**Fig. 6:**
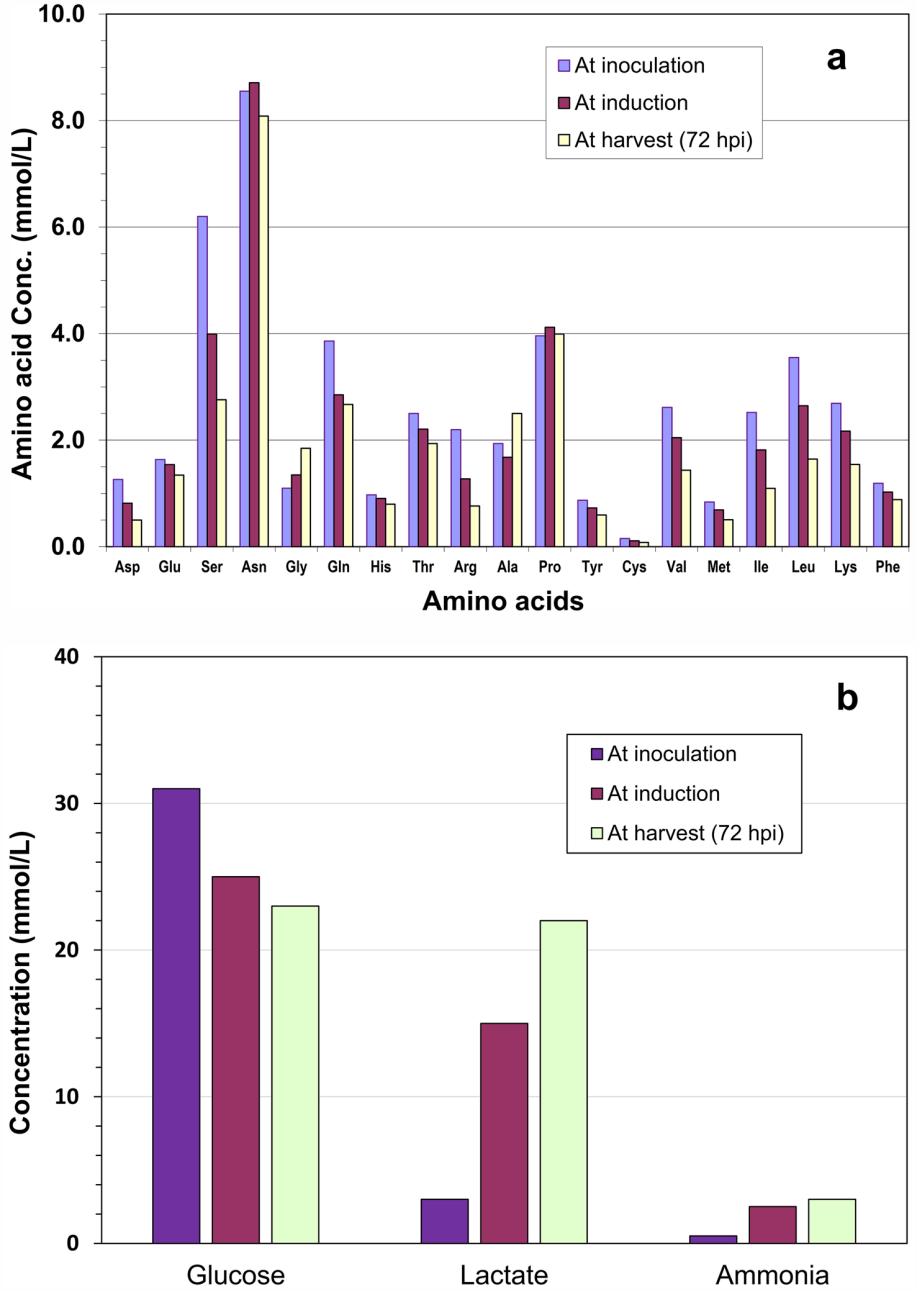
**a** Residual concentration of amino acids in samples taken from a culture grown in HyCell TransFx-H, fed with Cellboost 5 and induced with 5E6 cells/mL without medium exchange. **b** Concentration of glucose, lactate and ammonia over the time course of culture

Spent media from non-induced or induced cultures were analyzed for concentration of residual nutrients and inhibitory metabolites, typically amino acids, glucose, lactate and ammonia. Figure 6 depicts the residual concentration of amino acids, glucose, lactate and ammonia at inoculation, induction and 72 hpi in an exemplary culture fed with CB5 and induced at 5 × 10^6^ cells/mL without medium exchange. These results show that amino acids and glucose were not depleted while the concentration of lactate and ammonia was within a normal range, suggesting limiting nutrient(s) or inhibitory metabolite(s) were not the above monitored components. Feeding induced culture with individual component might not be an effective and fast approach to improve or optimize nutrient supply and to increase the virus production, considering complexity of culture media consisting of up to 100 components.

### Synergetic effect of mixing culture media and feed on growth of clone P/cSIN/92

**Fig. 7:**
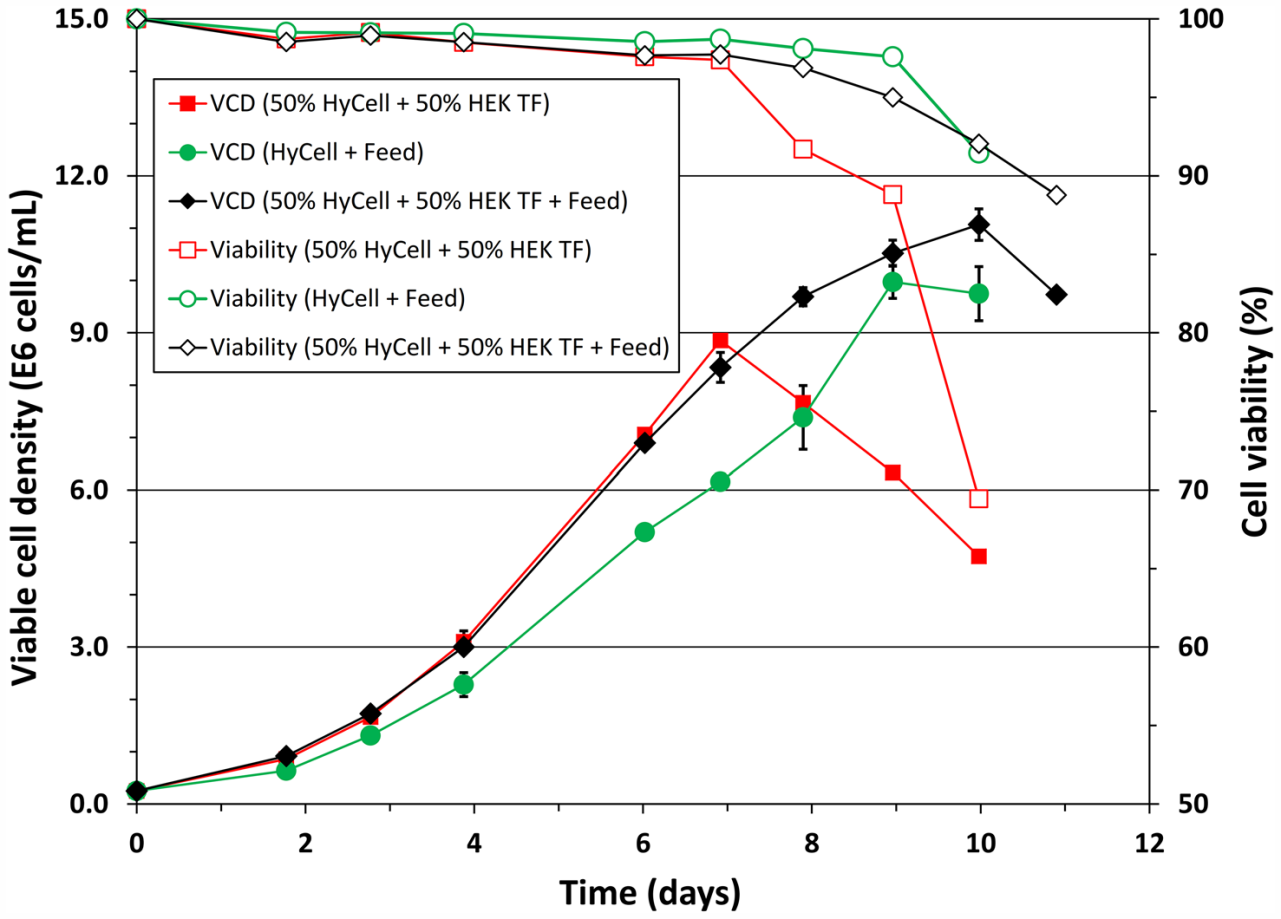
Growth curves of clone P/SIN/18 cultivated in a mixture of HyCell TransFx-H (HyCell) and HEK TF (red square) and also in the mixture (black diamond) or HyCell TransFx-H (green circle) fed with HEK FS feed. Bars represent the mean of duplicate cultures ± standard deviation (colour figure online)

The results in the previous sections show that HEK TF supported robust cell growth (Fig. 3), while the LV production was better when the cells were cultivated in HyCell TransFx-H (Fig. 4). Therefore HyCell TransFx-H was mixed with HEK TF or fed with HEK FS feed to broaden nutritional components and to exploit if there will be any synergetic or complementary effect between the media and feed from two different companies on the cell growth and also their potentials on the LV production in cultures induced at higher density. Data in Fig. 7 show that when the cells were cultured a mixture of HyCell TransFx-H and HEK TF (50% to 50%), the maximum viable cell density reached 8.85 × 10^6^ cells/mL, higher than the maximum viable cell density, respectively, achieved in the HyCell TransFx-H (5.76 × 10^6^ cells/mL) or HEK TF (8.11 × 10^6^ cells/mL). Feeding the cells cultivated in HyCell TransFx-H with HEK FS feed increased the maximum viable cell density from 5.76 × 10^6^ cells/mL to 9.97 × 10^6^ cells/mL, resulting in a cell density increment of 4.21 × 10^6^ cells/mL. The increment is much higher than the improvement of 0.68 × 10^6^ cells/mL observed in the HEK TF culture fed with HEK FS (Fig. 3). Furthermore, when the cells cultivated in a mixture of 50% HyCell TransFx-H and 50% HEK TF were fed with HEK FS, the maximum viable cell density further increased to 11.1 × 10^6^ cells/mL, the best cell density achieved in all conditions tested. The data in Figs. 7 clearly suggest a synergetic effect between the two media and also the feed in supporting the cell growth to high density.

### High titer production of LV in clone P/cSIN/92 cultivated with HyCell TransFx-H, HEK TF and HEK FS feed at high cell density

**Fig. 8:**
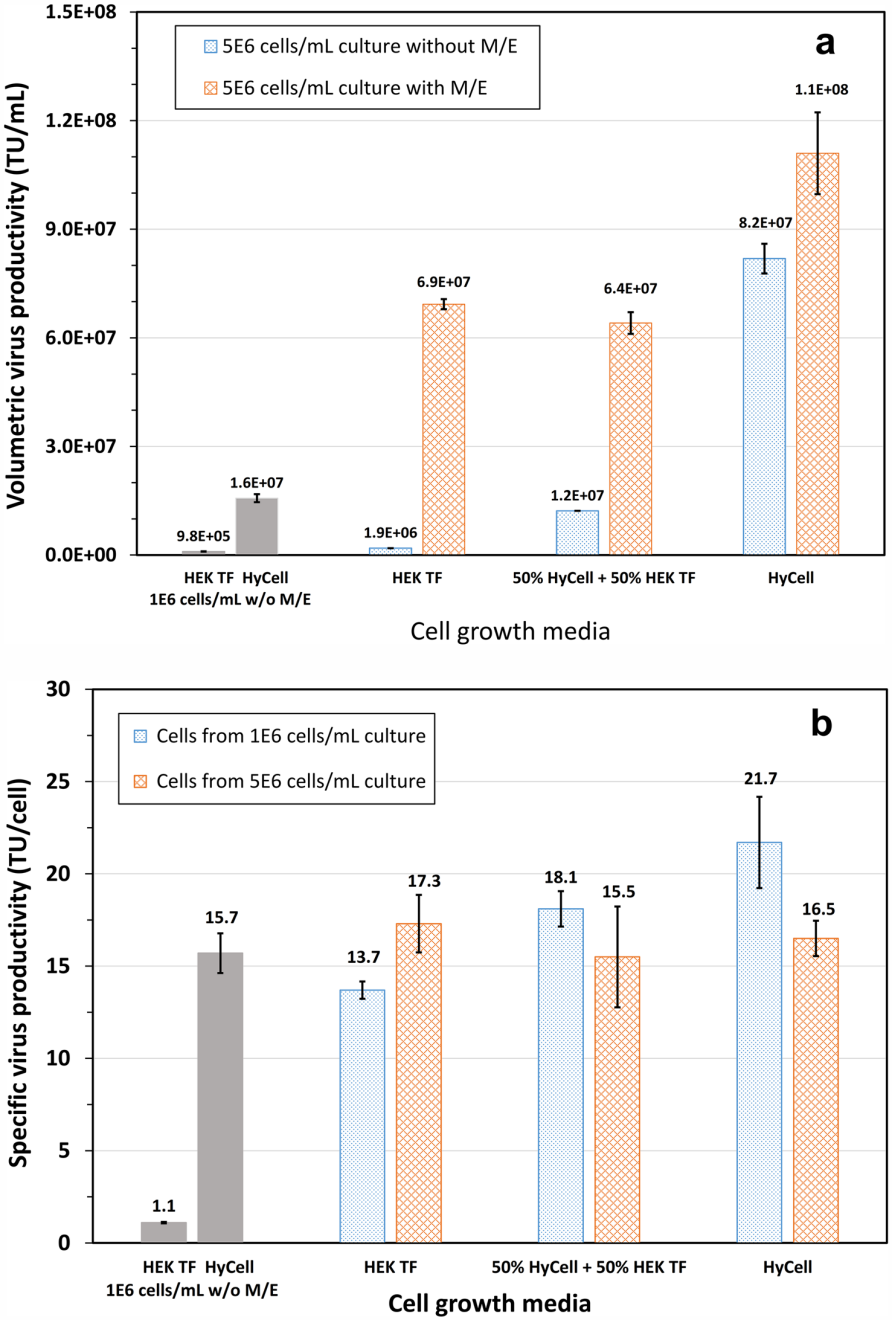
**a** Lentiviral vector production in clone P/cSIN/92 cultures grown in HEK TF, HyCell TransFx-H (HyCell), or a mixture of HyCell TransFx-H and HEK TF, and fed with HEK FS feed to a cell density of 5E6 cells/mL and induced with or without medium exchange to fresh HyCell TransFx-H. Two cultures (HEK TF and HyCell, 1E6 cells/mL w/o M/E) grown in HEKTF and HyCell TransFx-H (HyCell), respectively, to a cell density of 1E6 cells/mL and induced without medium exchange were conducted as references. **b** Cell-specific vector productivity of clone P/cSIN/92 taken from cultures grown in HEK TF, HyCell TransFx-H (HyCell), or a mixture of HyCell TransFx-H and HEK TF to cell density of 1E6 and 5E6 cells/mL (with HEK FS feed), then resuspended to fresh HyCell TransFx-H at 1E6 cells/mL prior to induction

Experimental design in this study took into consideration of previously obtained results (Figs. 3 and 7) and focused on effect of the following parameters on the LV productivity according to the scheme in Fig. 1: (i) growth medium, (ii) feed (HEK FS) used during the cell growth phase, (iii) medium exchange and (iv) cell density prior to induction. In these experiments, two cultures, respectively, grown in HEK TF and HyCell TransFx-H to a cell density of 1 × 10^6^ cells/mL and induced without medium exchange were added. HyCell TransFx-H culture was as a reference while HEK TF culture was used for comparison. The data in Fig. 8a revealed that the LV volumetric productivity in the HyCell TransFx-H reference was 1.57 × 10^7^ TU/mL while the titer from HEK TF culture was only 9.8 × 10^5^ TU/mL. The LV productivity increased to a titer range between 6.4 × 10^7^ and 11.1 × 10^7^ TU/mL when the cultures were induced at a cell density of 5 × 10^6^ cells/mL and with a medium exchange to fresh HyCell TransFx-H prior to the induction. This is a 4- to sevenfold increase compared to the titer in the HyCell TransFx-H reference culture. However, in the cultures induced at 5 × 10^6^ cells/mL, but without a medium exchange to fresh HyCell TransFx-H, such improvement in LV productivity was not retained in the cultures grown in HEK TF or a mixture of 50% HEK TF and 50% HyCell TransFx-H. Only the culture grown in HyCell TransFx-H retained a high titer at 8.2 × 10^7^ TU/mL, or fivefold improvement in the LV productivity even without a medium exchange. This result was very significant when compared to no more than twofolds of improvement obtained in the cultures using HyCell Transfix-H as growth media and fed with CB5 and other nutrients (Table 2). The fivefold improvement in the culture without a medium exchange before induction is also technically significant. This demonstrated that a procedure for medium exchange before induction is not necessary and implementation of this developed high cell density and high titer process is feasible at different scale bioreactors.

As illustrated in the experimental design in Fig. 1 for this set of experiment, the “quality” of the cells, respectively, grown in HyCell TransFx-H, HEK TF and a mixture of 50% HEK TF + 50% HyCell TransFx-H and fed with HEK FS was also assessed for supporting the LV production. The cells from various cultures (grown to 1 and 5 × 10^6^ cells/mL in different media) were resuspended to fresh HyCell TransFx-H at a cell density of 1 × 10^6^ cells/mL to have the same culture environment during the vector production phase. The data in Fig. 8b revealed the cell specific vector productivity was in a range of 14–22 TU/cell, and the difference was even smaller (between 15.5 and 17.3 TU/cell) among the cells from the cultures grown to 5 × 10^6^ cells/mL. This result once again suggests the low vector production was mainly due to the nutrient limitation in the cultures grown in HEK TF or a mixture of 50% HEK TF + 50% HyCell TransFx-H and induced at high cell density. However, similar to that presented in Fig. 6, analysis of spent media from these cultures suggested the cell growth or virus production was unlikely due to the nutrient limitation of amino acids or glucose, or inhibited by lactate and ammonia.

### Applicability of the developed feeding strategy to another stable clone for high titer production of LV at high cell density

**Fig. 9:**
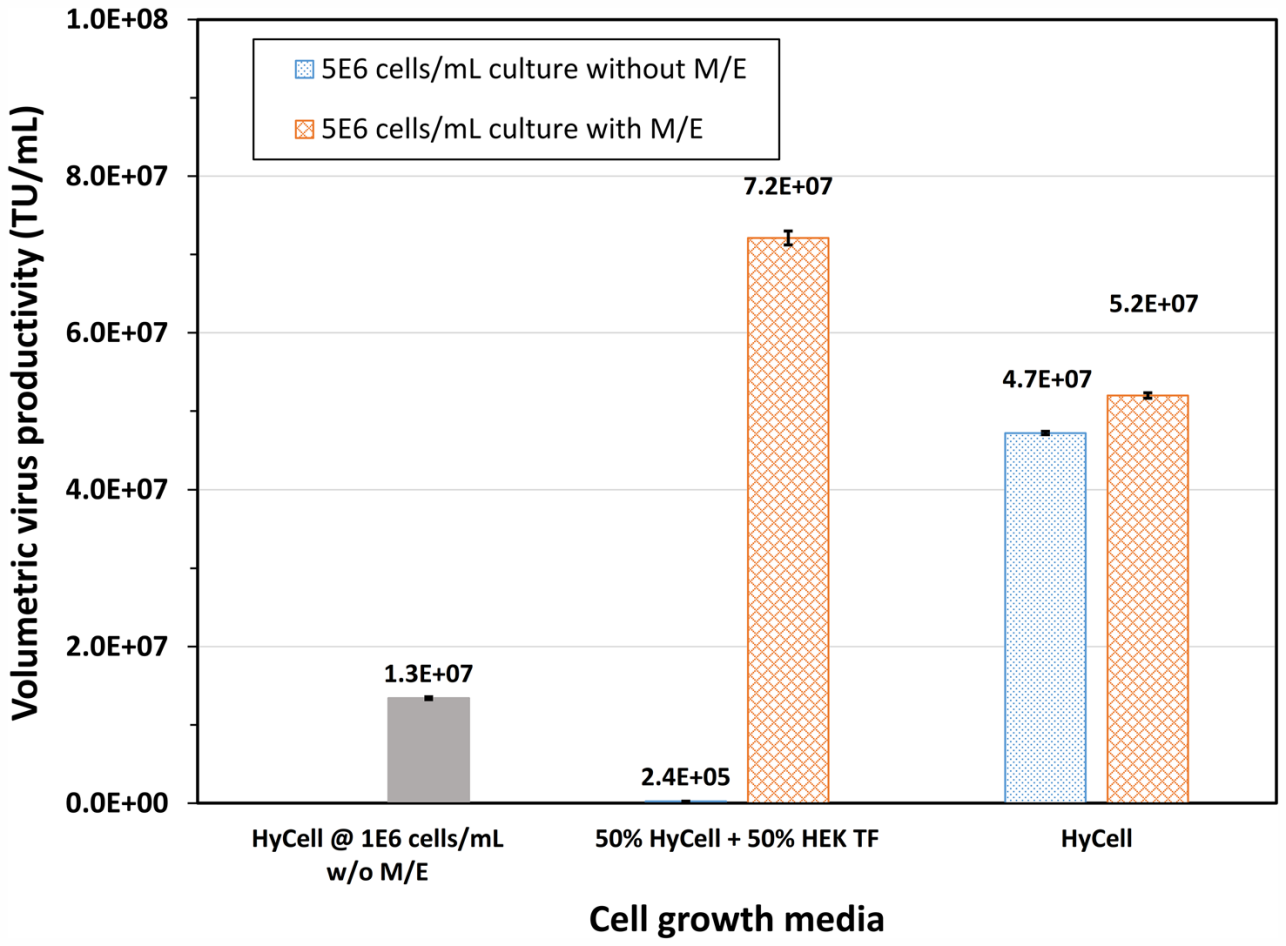
Lentiviral vector production in clone P/SIN/18 cultures grown in HyCell TransFx-H (HyCell), or a mixture of 50% HyCell TransFx-H and 50% HEK TF, and fed with HEK FS feed to a cell density of 5E6 cells/mL, and induced with or without medium exchange to fresh HyCell TransFx-H. A culture (HyCell @ 1E6 cells/mL w/o M/E) grown in HyCell TransFx-H (HyCell) to a cell density of 1E6 cells/mL and induced without medium exchange was conducted as reference

The optimal feeding strategy developed in the previous section, namely growing the clone in HyCell TransFx-H, feeding the culture with HEK FS feed and then inducing the culture at 5 × 10^6^ cells/mL without a medium exchange, was applied to clone P/SIN/18 for high titer production of LV. The clone P/SIN/18 was generated from HEK293 cell by a co-transfection, different from the transduction process used for P/cSIN/92. Data in Fig. 9 show the LV titer improved from 1.3 × 10^7^ to 4.7 × 10^7^ TU/mL, or a 3.5-fold improvement, when the cell density at induction was increased from 1 × 10^6^ to 5 × 10^6^ cells/mL in the cultures induced without medium exchange. However, it one again showed the high titer was only maintained in the culture grown in HyCell TransFx-H, suggesting the developed feeding strategy was able to provide nutrition requirements for two clones generated from HEK293 by different processes.

The results from this study demonstrated that the volumetric LV productivity could be improved by up to fivefold in shake flask cultures through nutrient optimization or balancing of culture media and development of feeding strategy. Although the conditions such as DO and pH in shake flask cultures were not controlled, oxygen supply (or DO concentration) was less likely to be a limiting factor for cultures induced at ≤ 5 × 10^6^ cells/mL, as a cell density up to 20 × 10^6^ cells/mL was achieved before in the shake flask culture conditions (agitation and culture volume) employed in this study. pH in the culture tended to decline with accumulation of lactate generated over the time course. Its effect on the LV production has not been tested yet. The high cell density process developed in this study was successfully scaled up to single bench scale (3L) bioreactor for high titer production of LV by other member in the team. Data will be published separately.

## Discussion

The stable producer clone P/cSIN/92 was generated from HEK293. No commercial media have been specifically designed or developed for this clone yet, although there are many different commercial media that have been developed and marketed for HEK293 cell line. Due to the relationship between clone P/cSIN/92 and HEK293, a total of six commercial HEK293 media from four different manufacturers were selected for evaluation in this study by hypothesizing the HEK293 media might also have a good probability in supporting robust growth of clone P/cSIN/92. The result from this study, however, shows that the clone P/cSIN/92 did not grow in FreeStyle F17. The cell density was only 2.7 × 10^6^ cells/mL, and 3.7 × 10^6^ cells/mL, respectively, in batch culture with BalanCD HEK293 or SFM4transfx 293, which is much lower than 8 × 10^6^ cells/mL and 5 × 10^6^ cells/mL, respectively, obtained in the above two media for HEK293 cells. In addition, although significant higher cell density (5.7 × 10^6^ to 8.1 × 10^6^ cells/mL) was achieved in HyCell TransFx-H, Xell HEK GM and HEK TF, these densities are still substantially lower than their respective density obtained in the parental HEK293 culture. These results suggest that the optimal nutritional requirement for clone P/cSIN/92 during the cell growth phase is different from what is required by parental HEK293 cell. The change in nutritional requirement is most likely due to the transfection of parental HEK293 cell with foreign DNA which was integrated into the cellular genome. As a result, the media designed for HEK293 cells might be not optimal anymore, and improvement of existing HEK293 media or development of new media might be needed for a robust growth of clone transfected from HEK293.

The maximum cell density increased dramatically when the clone was cultured in a mixture of 50% HyCell TransFx-H and 50% Xell HEK TF or in HyCell TransFx-H, and fed with HEK FS, indicating synergetic effect between the media and feed developed by two media companies, and also implying complex nutritional requirements by clone P/cSIN/92.

Despite the higher cell density achieved in the batch cultures using HyCell TransFx-H or HEK TF, the LV productivity was poor in the culture grown in HEK TF and only limited improvement (53%) was achieved in the batch cultures grown in HyCell TransFx-H medium and induced at higher cell density. This clearly shows that a culture medium, which is superior in supporting the growth of cell, does not necessary warrant a high vector productivity. This also suggests that, while high cell concentrations can usually be achieved by appropriate cultivation strategies, the corresponding cell‐specific vector yields often are difficult to achieve. This so called “cell density effect,” first described by Wood et al. [23], has been repeatedly used over the past years to describe lower than expected cell‐specific vector yields [22, 24–26]. This phenomenon may be explained by the biphasic processes of cell culture-derived viral and vector production which comprise an initial cell growth phase and a virus replication phase that initiates with inoculation by seed virus [27]. The nutritional requirement in the virus production phase may be different from what is needed during the cell growth phase.

The different nutritional requirements during the phase of cell growth and vector production were further exemplified by the vector productivity in the cultures using different basal media, fed with HEK FS feed and induced at a cell density of 5 × 10^6^ cells/mL with or without medium exchange to fresh HyCell TransFx-H (Fig. 8a). A high volumetric vector productivity was achieved in all three culture conditions induced with medium exchange. However, when the cultures were induced without medium exchange, a high titer (8.19 × 10^7^ TU/mL) was only maintained in the culture grown in HyCell TransFx-H and fed with HEK FS feed. The vector production was 1.93 × 10^6^ TU/mL or 42 times lower when HEK TF was used as a basal growth medium and also fed with HEK FS. This may suggest that some nutrients in HyCell TransFx-H, but not in HEK TF, are required during the phase of LV production. Cell-specific vector productivity of clone P/cSIN/92 from cultures grown to 1 × 10^6^ or 5 × 10^6^ cells/mL in the three different media (Fig. 8b) was in a range of 14–21 TU/cell, relatively constant. This suggests the lower LV productivity is more likely due to nutrient limitation in the cultures induced without medium exchange.

The clone P/cSIN/92 grown in HyCell TransFx-H was, respectively, fed with CB5, yeast extract and/or insulin to improve the LV productivity through induction at high cell density. The feeding strategy was similar to what was used in the HyCell TransFx-H/HEK FS process; however, the improvement in LV productivity was only up to 21%. This result may suggest that supplementing batch cultures with individual component might not be an effective or fast approach to improve the performance of culture in terms of cell growth and/or virus production, as most commercial culture media are proprietary and their formulation is complex (consisting of up to 100 components). A combination of culture media and/or feed, especially from different manufacturers, will more likely provide a much broader range of nutrients to meet variable nutritional requirements of a new clone and could be a fast approach to improve the cell growth and virus production.

The titer of 8.19 × 10^7^ TU/mL achieved in the culture grown in HyCell TransFx-H, fed with HEK FS feed and induced without medium exchange is very promising, higher than most of the titers reported by others either in adherent or suspension cultures. These reported titers are in a range of 10^6^ to 5 × 10^7^ TU/mL as summarized in review articles [7, 9, 16]. The titer of 8.19 × 10^7^ TU/mL titer is comparable to the cumulative titers of 5 to 8 × 10^7^ TU/mL achieved in perfusion culture [18]. Recently titers at 2 × 10^8^ TU/mL have been reported through adaptation of HEK293T to grow in suspension culture and transfected under optimized conditions [13].

Batch culture fed with nutrients (fed-batch) is widely used for productions of biopharmaceuticals, especially antibody production by CHO culture in which final MAb titer increases with cell density and longevity of cell culture [19]. In comparison, development of feeding strategy to improve production of viruses and viral vectors was only sporadically reported [21]. Liu et al. [28] employed a feeding strategy to enhance rAAV production in the baculovirus/insect cell system and resulted in a 2.6-fold higher when the cultures in both batch and fed-batch systems were infected at 1 × 10^6^ cells/mL. A sevenfold improvement in AAV titer was reported when the culture was infected at higher cell density (5 × 10^6^ cells/mL) and with optimized MOI and fed after infection [29]. While Lee et al. [30] was able to improve the titer of adenovirus by tenfold by controlling the glutamine concentration at a low level with a concentrated glucose-free feed medium and infecting the culture at 3 × 10^6^ cells/mL in comparison to the batch culture infected at 1.5 × 10^6^ cells/mL. In these reported works, it is not clear if the volumetric virus productivity was improved by the increased cell density at infection and/or by the cell-specific virus productivity. Recently, our team has explored the potential of perfusion culture for high titer production of lentiviral vector and achieved up to 15 times of improvement in titer through induction at a cell density ≥ 5 × 10^6^ cells/mL [18]. However, the operation of perfusion process is costly and more complicated than batch or fed-batch process.

The result from this study revealed that clones P/cSIN/92 and P/SIN/18 (stably transfected HEK293 cell) and its parental cell line, HEK293, may possess diverse nutritional requirements that are unique to each clone or cell line. Universal serum-free media applicable to all cell lines are less likely available. The complexity of culture media selection/improvement and feeding strategy development for high titer LV production at high cell density is compounded by different nutritional needs in the biphasic processes of cell culture-derived viral and vector production. Improvement of a single parameter (such as basal medium or cell density for example) was insufficient to improve the overall process outcome. Developmental efforts on the other parameters were required. In our current process, optimization of three parameters (basal medium, feed and cell density) was necessary to realize the maximal production potential of LV. The developed process, which offers high LV productivity and does not require medium exchange before induction, is easily scalable and could be a potential process as a cost-effective and simple platform for high titer production of LV at high cell density.
